# Racial and Socioeconomic Disparities Are More Pronounced in Inflammatory Breast Cancer Than Other Breast Cancers

**DOI:** 10.1155/2017/7574946

**Published:** 2017-08-15

**Authors:** Ryan A. Denu, John M. Hampton, Adam Currey, Roger T. Anderson, Rosemary D. Cress, Steven T. Fleming, Joseph Lipscomb, Xiao-Cheng Wu, J. Frank Wilson, Amy Trentham-Dietz

**Affiliations:** ^1^Medical Scientist Training Program, University of Wisconsin, 6068 WIMR, 1111 Highland Avenue, Madison, WI 53705, USA; ^2^University of Wisconsin Carbone Cancer Center, 610 Walnut Street, Room 307, WARF Building, Madison, WI 53726, USA; ^3^Department of Radiation Oncology, Medical College of Wisconsin, 8701 W Watertown Plank Rd, Milwaukee, WI 53226, USA; ^4^University of Virginia School of Medicine, West Complex MSB, Room 6203E, 1300 Jefferson Park Ave, Charlottesville, VA 22908, USA; ^5^Public Health Institute, Cancer Registry of Greater California, Department of Public Health Sciences, UC Davis School of Medicine, 4610 X St, Davis, CA 95817, USA; ^6^College of Public Health, University of Kentucky, 111 Washington Avenue, Lexington, KY 40536, USA; ^7^Rollins School of Public Health and Winship Cancer Institute, Emory University, 1518 Clifton Road, Atlanta, GA 30322, USA; ^8^LSU Health Sciences Center School of Public Health, 2020 Gravier Street, 3rd Floor, New Orleans, LA 70112, USA; ^9^Department of Population Health Sciences, University of Wisconsin Carbone Cancer Center, 610 Walnut Street, Room 307, WARF Building, Madison, WI 53726, USA

## Abstract

Inflammatory breast cancer (IBC) is a rare yet aggressive form of breast cancer. We examined differences in patient demographics and outcomes in IBC compared to locally advanced breast cancer (LABC) and all other breast cancer patients from the Breast and Prostate Cancer Data Quality and Patterns of Care Study (POC-BP), containing information from cancer registries in seven states. Out of 7,624 cases of invasive carcinoma, IBC and LABC accounted for 2.2% (*N* = 170) and 4.9% (*N* = 375), respectively. IBC patients were more likely to have a higher number (*P* = 0.03) and severity (*P* = 0.01) of comorbidities than other breast cancer patients. Among IBC patients, a higher percentage of patients with metastatic disease versus nonmetastatic disease were black, on Medicaid, and from areas of higher poverty and more urban areas. Black and Hispanic IBC patients had worse overall and breast cancer-specific survival than white patients; moreover, IBC patients with Medicaid, patients from urban areas, and patients from areas of higher poverty and lower education had worse outcomes. These data highlight the effects of disparities in race and socioeconomic status on the incidence of IBC as well as IBC outcomes. Further work is needed to reveal the causes behind these disparities and methods to improve IBC outcomes.

## 1. Background

Inflammatory breast cancer (IBC) is a relatively rare, yet aggressive, subtype of locally advanced breast cancer, with median overall survival less than 4 years [1, 2]. IBC incidence ranges from 1 to 6% of all breast cancers (most recent data suggests 2.5%) among women in the United States, yet they account for 7% of all breast cancer deaths [3–9]. Approximately 25% of IBC patients present with distant disease at diagnosis [4]. IBC was historically a uniformly fatal disease, but the implementation of multifactorial treatments with neoadjuvant chemotherapy, surgery, and radiation has improved survival over the past two decades [10–13]. Response to chemotherapy is one of the strongest predictors of survival [14]. Nevertheless, many patients experience disease recurrence, most frequently to bone, brain, lung, or liver [15].

With regard to patient characteristics, IBC patients tend to be younger than other breast cancer patients, with a median age at diagnosis of 57 years for IBC compared to 61.9 for all breast cancers combined [5, 6]. IBC has been shown to have a higher incidence rate among black women than white women (3.1 per 100,000 women-years for blacks compared to 2.2 for whites) [3]. Further, overall survival has been reported to be significantly worse for blacks than whites [6]. High BMI is associated with an increased risk for IBC compared to non-IBC breast cancer [16, 17].

Survival tends to be poorer in IBC patients compared to non-IBC breast cancer patients regardless of histologic subtype [18, 19]. Data from the National Cancer Institute's Surveillance, Epidemiology, and End Results (SEER) Program indicate that the 5-year survival rate was 34% for IBC patients from 1988 to 2001 compared to about 87% for other invasive breast cancers [20]. Although survival for cases of IBC has improved with multimodal therapy [21], recent population statistics still demonstrate much lower median survival for IBC cases (2.9 years) than for cases of locally advanced breast cancer (6.4 years) and nonmetastatic breast cancer (>10 years). Therefore, there is great need to identify factors impacting survival in IBC, and the objective of this study was to identify those factors. Using a multistate, population-based sample of patients with IBC, we examined the demographics of these IBC patients compared to patients with locally advanced breast cancer (LABC) because IBC was earlier thought to be a more aggressive form of LABC [10], and LABC has been used frequently as a comparison group for IBC [22]. Further, we examined differences in IBC patient survival based on patient characteristics.

## 2. Materials and Methods

### 2.1. Patient Population and Data Sources

The Breast and Prostate Cancer Data Quality and Patterns of Care Study (POC-BP) is the third and most comprehensive POC study from the National Program of Cancer Registries of the Centers for Disease Control and Prevention (CDC). It collected information on breast and prostate cancer patients diagnosed in 2004 in seven states (California, Georgia, Kentucky, Louisiana, Minnesota, North Carolina, and Wisconsin). Cancer registry data were supplemented by reabstracting hospital records and obtaining information about adjuvant treatment and comorbidity from physicians and outpatient facilities and linkages with secondary files such as census data or hospital/physician files. The combined sample size was 9,142 breast cancer patients, from which we extracted patients with IBC (*n* = 170) and compared them to patients with LABC (*n* = 375). Our IBC population was defined as T4d, N0-2, and M0-1. LABC was defined as a diagnosis of stage IIIB or IIIC (this does not include stage III not otherwise specified). As another comparison group, “all else,” we included all 9,142 breast cancer patients minus in situ cases and those classified as IBC or LABC (*n* = 7,079).

### 2.2. Covariates of Interest

Patient race and ethnicity were attained from medical records and categorized into the following groups: non-Hispanic white, non-Hispanic black, Hispanic, and others. Insurance status at diagnosis was categorized into the following five groups: private, Medicare/other public, Medicaid, none, and unknown. The private insurance group also included cases where the patient had both Medicare and private insurance. “Other public insurance” consisted of patients with TRICARE, other military insurance, Veterans Affairs, or Indian Health Service coverage. The Medicaid group also included women on Medicare with Medicaid eligibility and other government programs. Body mass index (BMI) at the time of diagnosis was categorized into three groups: <25 kg/m^2^ (normal), 25–29 kg/m^2^ (overweight), and ≥30 kg/m^2^ (obese). Area measures were constructed from 2000 US census data linked to the census tract of the patient's residence at the time of diagnosis.

Hospital/institution characteristics were based on the facility in which the patient received breast cancer surgery regardless of the location of other treatments because most referrals for adjuvant therapy are made by a surgeon [23]. If the patient did not receive surgery, then we used the institution where the patient received chemotherapy. If the patient did not receive chemotherapy, then we used the institution where the patient received endocrine therapy.

Patient comorbidities were assessed using the Adult Comorbidity Evaluation-27 (ACE-27) developed by Piccirillo et al. [24]. The ACE-27 was chosen because it reflects a wide range of coexisting conditions and disease severity relevant to cancer therapy choice and outcome.

### 2.3. Statistical Analysis

Chi-square tests were used to compare IBC to LABC and all other breast cancer patients. Kaplan-Meier survival curves were drawn to compare patients according to different characteristics, with follow-up beginning at the date of diagnosis through either death or censoring, whichever occurred first. Log-rank tests were calculated to compare survival curves. Cox proportional hazards modeling was used to assess the predictive value of certain factors on the survival of IBC patients. All significance tests were two-sided, and *P* values less than 0.05 were considered statistically significant. All statistics were weighted by the sampling fractions used by each state registry for the respective sampling stratum to represent the source population. SAS procedures for survey data were used.

## 3. Results

### 3.1. Demographic and Clinical Characteristics of IBC Patients versus LABC Patients

We assessed differences in demographics of the patients as well as molecular and clinical characteristics of the disease in IBC compared to LABC and all other breast cancers (Table 1). The mean age of IBC patients was 57.7 years compared to 58.9 for LABC and 59.5 for all others. IBC patients were more likely to be of black race than other breast cancer patients. A greater percentage of IBC patients were obese (BMI > 30) compared to other breast cancer patients. IBC patients were more likely to have comorbidities than other breast cancer patients, and the severity of those comorbidities was also greater for IBC patients compared to other breast cancer patients; no differences in comorbidity number or severity were observed between IBC and LABC patients. IBC patients were more likely to be diagnosed by signs or symptoms other than a lump compared to LABC and all other breast cancer patients. In addition, IBC patients were more likely to be from areas with higher poverty compared to other breast cancer patients, but there was no significant difference compared to LABC patients (Table 2). IBC patients were more likely to be treated at large (>400 beds), Commission on Cancer-accredited, teaching hospitals compared to LABC and all other patients.

### 3.2. Clinical Metastatic versus Nonmetastatic IBC Patient Characteristics

We assessed differences in patient characteristics in IBC patients that presented with clinical metastatic disease (*n* = 63; 37%) versus nonmetastatic disease (*n* = 107; see Supplemental Table 1 in the Supplementary Material available online at https://doi.org/10.1155/2017/7574946). There were no differences in age between metastatic and nonmetastatic IBC patients. Patients presenting with metastatic disease were more likely to be of black race, be insured by Medicaid, and be from areas of higher poverty and more urban areas.

### 3.3. Factors Predicting Survival of IBC Patients

**Figure 1:**
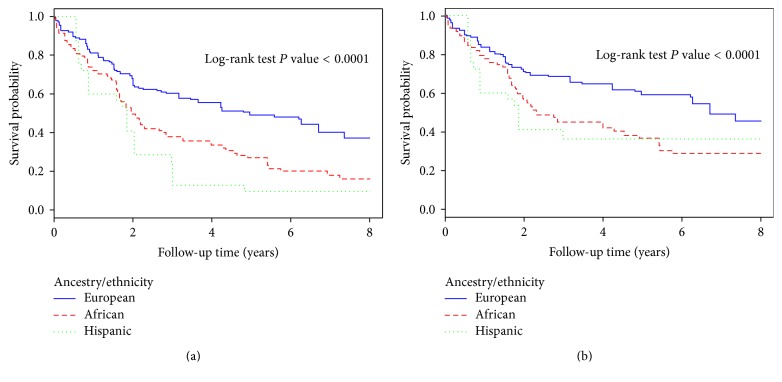
All-cause overall survival (a) and breast cancer-related survival (b) among inflammatory breast cancer patients based on race and ethnicity, Patterns of Care, 2004–2012.

We compared all-cause and breast cancer-related survival of IBC patients to LABC and all other breast cancer patients. IBC and LABC patients both had significantly worse survival compared to all other breast cancer patients, and IBC patients had significantly worse survival compared to LABC patients (both all-cause and breast cancer-related survival; Supplemental Figure 1). IBC patients of black race and Hispanic ethnicity had worse all-cause and breast cancer-related survival compared to white IBC patients (Figure 1). We also compared survival based on ER/PR status, finding that IBC patients with ER/PR-positive tumors had better all-cause and breast cancer-specific survival (Supplemental Figure 2). Lastly, IBC patients who presented with metastatic disease had significantly worse all-cause and breast cancer-related survival compared to nonmetastatic (Supplemental Figure 3).

Differences in IBC patient survival were also analyzed based on patient characteristics using Cox proportional hazards modeling (Table 3). All Cox models were adjusted for age and metastasis on presentation and weighted based on sampling. Black and Hispanic IBC patients had worse survival compared to white IBC patients; this disparity was not observed in LABC patients. IBC patients with BMI < 25 had 5-year survival rates of 68.5% compared to 41.6% for overweight (BMI 25–29) patients and 64.7% for obese patients (BMI ≥ 30); there were no differences in survival rates of LABC patients based on BMI. Higher number and severity of comorbidities correlated with worse survival rates, both for IBC and for LABC. IBC patients with Medicaid had worse survival than IBC patients with private insurance or Medicare; the same trend was observed in LABC. Furthermore, IBC patients from areas of higher poverty and lower education had worse survival rates, but this was not the case for LABC patients. Patients with metastatic disease on presentation and with ER− and PR− negative tumors had worse survival. Survival was worse in patients with poorly differentiated tumors compared to well or moderately differentiated tumors.

## 4. Discussion

IBC is a rare yet lethal form of LABC. Epidemiological research on IBC has been lacking due to a low incidence of the disease, and most data regarding outcomes of IBC patients are from small single institution series. This Patterns of Care Study with patients from 7 states compared IBC to LABC and all other breast cancers in patient and tumor characteristics and survival. We found that IBC patients had significantly worse survival than LABC and other breast cancer patients. Our data revealed a 5-year survival rate of 44% for IBC compared to 58% for LABC and 84% for all other cases. This is consistent with other studies [25–27]. Despite the advent of multimodal therapy, including both neoadjuvant and adjuvant chemotherapy, IBC continues to carry a poor prognosis.

The most striking findings in this dataset are the variations in risk and severity of IBC cases based on race and socioeconomic status. A higher percentage of black breast cancer patients have IBC compared to white breast cancer patients. Previous studies have demonstrated similar racial disparities in IBC [3, 4, 28–31]. Furthermore, IBC patients were more likely to be from areas of higher poverty compared to other breast cancer patients. IBC patients presenting with metastatic disease were more likely to be of black race and from poorer, more urban areas. With regard to survival, we found that patients from areas of higher poverty and lower education had worse survival rates. In addition, black and Hispanic IBC patients had worse survival compared to white IBC patients. Consistent with this, white race has been shown to be an independent predictor of better survival in IBC [32]. It is important for physicians to be cognizant of these disparities so that the causes of such disparities can be better addressed.

The major potential reasons for this disparity in IBC incidence and outcomes based on race and socioeconomic status include (1) decreased access to care; (2) differences in biology due to race or heritage; (3) increased medical mistrust among minorities [33]; and (4) the erythema associated with IBC being less visible in patients with darker skin, potentially leading to a delay in diagnosis [34]. Based on our data and others [35, 36], we conclude that the first explanation is most justified; that is, black IBC patients have worse outcomes because they have reduced access to care, not because of biological differences based on race or heritage. Because race/ethnicity and socioeconomic status are so strongly correlated, black patients are less likely to get adequate breast cancer screening, and after diagnosis they generally have more limited access to care, limited treatment options, and difficulty with treatment adherence [37]. Also supporting this claim, Tunisia had a high rate of IBC which has decreased at least in part due to improved socioeconomic status [38].

In our dataset, only 3.8% of IBC tumors were found by screening mammography. Many patients were diagnosed as a result of symptoms other than a lump. This is consistent with previous reports [15]. There are three hypotheses that could explain this finding. First, it is possible that these women were not getting regular screening mammograms. Secondly, it may be due to the aggressive nature of IBC, suggesting that these tumors developed rapidly between screenings. Lastly, it may be because mammography itself is less effective at detecting these tumors. Some reports have suggested that IBC is difficult to detect using mammography or clinical breast exam because there is often no mass [2]. When there is a mass, it is often masked by diffusely increased breast density. IBC is found in a disproportionate number of women of low socioeconomic status, and breast cancer screening is generally lower in people of lower socioeconomic status [39].

IBC patients tend to have worse comorbidities than other breast cancer patients. One study found that women with endocrine disease, psychiatric disorders, and hematologic disease were more likely to be diagnosed with advanced breast cancer, while those with cardiovascular disease, musculoskeletal disorders, gastrointestinal disease, benign breast disease, and genitourinary disorders were less likely to be diagnosed with advanced breast cancer after controlling for number of physician visits [40]. The risk for death among breast cancer patients depends heavily on the stage of illness at diagnosis, and comorbid illnesses are thought to increase the risk of late stage disease for a number of reasons [40, 41]. Some suggest that early symptoms of breast cancer are sometimes confused with the symptoms of comorbid illnesses or are less significant than other health problems [42]. Women with comorbidities are more likely to access health care and therefore may be more likely to get screened for breast cancer [43]. However, some studies suggest that comorbidities can be a barrier to breast cancer screening [44]; our data tends to support this hypothesis. Lastly, there may be some interaction between certain comorbidities and the development of cancer; for example, diabetes is thought to increase risk of cancer via hyperinsulinemia, hyperglycemia, and inflammation [45].

A major strength of this study is its large-scale population-based design, including data from seven states. Other strengths were the inclusion of National Program of Cancer Registries that did not participate in previous POC studies and comprehensive treatment information. Limitations include the small number of IBC cases, as all past IBC research has been hampered by this as well.

## 5. Conclusion

In conclusion, this study highlights potential reasons for disparities in IBC incidence and severity, which represent targetable—albeit not modifiable—factors. We identify disparities in race and socioeconomic status on the risk of IBC as well as IBC outcomes. Further work is needed to reveal the causes behind these disparities and methods to improve IBC outcomes.

## Supplementary Material

Supplemental Table 1: Characteristics of patients with inflammatory breast cancer according to presentation with metastatic versus non-metastatic disease, Patterns of Care Study, 2004-2012.
Supplemental Figure 1: Survival analysis of IBC patients compared to LABC and all other breast cancer patients based on all-cause (A) and breast cancer-related (B) mortality.
Supplemental Figure 2: Comparison of survival among IBC patients based on ER/PR status. Analysis was done based on all cause (A) and breast cancer-related (B) overall survival.
Supplemental Figure 3: Survival among IBC patients based on presentation with metastatic versus non-metastatic disease. Analysis was done based on all cause (A) and breast cancer-related (B) overall survival.

## Figures and Tables

**Table 1:** Demographic characteristics of patients with inflammatory breast cancer compared with locally-advanced and all other breast cancer patients, Patterns of Care Study, 2004–2012.

Characteristic	IBC		LABC (IIIB, C)		IBC versus LABC *P* value^*∗∗*^	Others		IBC versus others *P* value^*∗∗*^
	Number	%^*∗*^	Number	%^*∗*^		Number	%^*∗*^	
Total	170		375			7079		
Age at diagnosis, years								
<50	50	26.4	113	32.2	0.02	2024	26.0	0.09
50–59	49	34.1	91	21.3		1818	25.9	
60–69	36	22.6	76	18.8		1448	21.1	
≥70	35	16.9	95	27.6		1789	27.0	
Ancestry/ethnicity								
European, non-Hispanic	81	68.4	174	66.4	0.54	4083	76.4	0.002
African, non-Hispanic	68	22.4	138	21.6		1925	13.7	
Hispanic	14	7.6	38	8.2		615	6.2	
Other	7	1.6	25	3.9		456	3.8	
Body mass index (kg/m^2^)								
<25	28	16.2	88	25.5	0.28	1743	27.0	0.05
25–29	35	24.4	77	22.1		1626	23.5	
≥30	66	34.9	133	34.1		2086	27.6	
Unknown	41	24.4	77	18.4		1624	21.9	
Number of comorbidities								
0	58	34.8	154	42.8	0.08	3162	45.3	0.03
1	57	34.3	105	27.5		2028	28.7	
2	35	23.2	61	16.5		1127	15.2	
≥3	20	7.7	55	13.2		762	10.8	
Piccirillo Comorbidity Score								
None	55	33.0	146	41.0	0.53	2948	42.1	0.01
Mild	68	40.6	147	37.5		2957	41.5	
Moderate	29	17.0	49	14.1		709	10.0	
Severe	16	7.9	27	6.0		287	3.8	
Unknown	2	1.6	6	1.4		178	2.6	
Method of detection								
Screening mammography	8	3.8	72	19.9	<0.0001	3035	44.0	<0.0001
Clinical or self-breast exam	22	9.1	60	16.4		1055	13.7	
Self-detection of lump	58	43.3	163	44.2		2119	29.9	
Self-detection, no lump	74	37.1	62	14.6		399	5.2	
Other, unknown	8	6.7	18	4.9		471	7.1	
Patient health insurance								
Private	77	48.4	167	52.3	0.58	4077	60.5	0.0004
Medicare only/public	38	21.3	88	24.5		1553	23.0	
Medicaid	39	20.5	84	15.4		999	10.3	
No insurance or unknown	16	9.9	36	7.8		450	6.2	

^*∗*^Percentages weighted based on sampling design. ^*∗∗*^*P* values from chi-square tests.

**Table 2:** Contextual characteristics of patients with inflammatory breast cancer compared with locally advanced and all other breast cancer patients, Patterns of Care Study, 2004–2012.

Characteristic	IBC		LABC (IIIB, C)		IBC versus LABC *P* value^*∗∗*^	All else		IBC versus all else *P* value^*∗∗*^
	Number	%^*∗*^	Number	%^*∗*^		Number	%^*∗*^	
Total	170		375			7079		
Poverty (census tract)								
<20% below poverty	108	73.8	257	78.1	0.33	5280	82.3	0.01
≥20% below poverty	60	25.6	117	21.4		1779	17.4	
Education (census tract)								
<25% no high school degree	83	60.2	200	63.1	0.61	4297	68.5	0.07
≥25% no high school degree	85	39.2	174	36.3		2762	31.2	
Urbanicity								
Urban	94	53.8	185	47.8	0.14	3585	51.3	0.43
Rural	23	9.2	63	17.0		1014	13.4	
Urban/rural mix	51	36.5	126	34.7		2461	35.0	
Unknown	2	0.5	1	0.5		19	0.3	
Hospital teaching status								
Teaching	73	45.8	136	37.0	0.06	2807	39.2	0.10
Other	66	38.2	202	52.1		3330	47.5	
Unknown	31	15.9	37	10.9		942	13.3	
Hospital size (number of beds)								
<200	32	15.1	96	23.3	0.05	1671	21.2	0.17
200–299	20	12.4	77	23.0		1234	18.1	
300–399	21	14.7	38	11.4		842	13.2	
≥400	66	41.8	127	31.4		2390	34.1	
Unknown	31	15.9	37	10.9		942	13.3	
Hospital Commission on Cancer Accreditation								
No	59	29.3	152	40.0	0.06	2654	38.1	0.05
Yes	87	57.8	191	50.1		3558	49.1	
Unknown	24	12.9	32	9.9		867	12.7	

^*∗*^Percentages weighted based on sampling design. ^*∗∗*^*P* values from chi-square tests.

**Table 3:** Hazard ratios for inflammatory breast cancer patient mortality based on patient characteristics, Patterns of Care Study, 2004–2012.

Characteristic	At risk		Cause of death			
			Breast cancer		Any cause	
	Number	%^*∗*^	Number of deaths	HR (95% CI)^*∗∗*^	Number of deaths	HR (95% CI)^*∗∗*^
Total	170		91		121	
Age at diagnosis, years						
<50	50	26.4	25	1 (ref.)	30	1 (ref.)
50–59	49	34.1	31	**2**.**05** (**1**.**03**–**4**.**08**)	37	**1**.**89** (**1**.**03**–**3**.**47**)
60–69	36	22.6	18	1.15 (0.48–2.74)	24	1.28 (0.65–2.53)
≥70	35	16.9	17	1.92 (0.81–4.56)	30	**2**.**53** (**1**.**34**–**4**.**77**)
Ancestry/ethnicity						
European, non-Hispanic	81	68.4	36	1 (ref.)	48	1 (ref.)
African, non-Hispanic	68	22.4	43	**1**.**76** (**1**.**05**–**2**.**97**)	56	**1**.**86** (**1**.**19**–**2**.**91**)
Hispanic	14	7.6	8	1.14 (0.35–3.71)	11	1.57 (0.72–3.40)
Other	7	1.6	4	1.25 (0.67–2.33)	6	1.66 (0.88–3.13)
Piccirillo Comorbidity Score						
None	55	33.0	26	1 (ref.)	35	1 (ref.)
Mild	68	40.6	38	0.55 (0.27–1.12)	48	**0**.**48** (**0**.**27**–**0**.**87**)
Moderate	29	17.0	14	1.01 (0.43–2.41)	21	0.86 (0.39–1.86)
Severe	16	7.9	13	**2**.**77** (**1**.**35**–**5**.**69**)	16	**2**.**64** (**1**.**32**–**5**.**27**)
Unknown	2	1.6	0	—	1	0.88 (0.44–1.75)
Metastasis on presentation						
Yes	63	31.1	49	**3**.**20** (**1.87**–**5**.**48**)	59	**2**.**97** (**1**.**86**–**4**.**73**)
No	107	68.9	42	1 (ref.)	62	1 (ref.)
ER/PR status						
ER+ and/or PR+	87	49.1	38	0.55 (0.29–1.03)	56	**0**.**54** (**0**.**32**–**0**.**92**)
ER− and PR−	70	42.9	45	1 (ref.)	54	1 (ref.)
Unknown	13	8.1	8	2.11 (0.76–5.83)	11	1.89 (0.71–5.05)
HER2 status						
Positive	62	35.9	33	0.74 (0.40–1.36)	42	0.90 (0.55–1.49)
Negative	83	48.2	45	1 (ref.)	61	1 (ref.)
Unknown	25	16.0	13	0.84 (0.19–3.70)	18	0.80 (0.21–3.07)
Grade						
Well or moderately differentiated	31	22.4	13	1 (ref.)	21	1 (ref.)
Poorly differentiated	112	66.0	64	1.84 (0.72–4.71)	84	1.83 (0.91–3.69)
Undifferentiated	6	1.8	2	0.46 (0.05–3.91)	2	0.38 (0.05–2.81)
Unknown	21	9.9	12	1.12 (0.39–3.20)	14	0.84 (0.36–1.96)

ER, estrogen receptor; HR, hazard ratio; PR, progesterone receptor; ref., reference group. ^*∗*^Percentages weighted based on sampling design. ^*∗∗*^Cox proportional hazards models adjusted for age, metastasis on presentation, ER/PR status, treatment, and sampling weights and strata within each study center.
