# Supplementary material for: Racial and Socioeconomic Disparities Are More Pronounced in Inflammatory Breast Cancer Than Other Breast Cancers
Source: J Cancer Epidemiol. 2017 Aug 15;2017:7574946. doi: 10.1155/2017/7574946 (PMC5574219; doi:10.1155/2017/7574946)

**Supplemental Table 1.** Characteristics of patients with inflammatory breast cancer according to presentation with metastatic versus non-metastatic disease, Patterns of Care Study, 2004-2012.

|  | **IBC metastatic** | | **IBC not-metastatic** | |  |
| --- | --- | --- | --- | --- | --- |
| **Characteristic** | **Number** | **%*** | **Number** | **%*** | **P value**** |
| Total | 63 |  | 107 |  |  |
| Age at diagnosis, years |  |  |  |  |  |
| <40 | 4 | 6.2 | 13 | 7.2 | 0.93 |
| 40-49 | 13 | 17.1 | 20 | 20.6 |  |
| 50-59 | 18 | 37.4 | 31 | 32.6 |  |
| 60-69 | 16 | 22.8 | 20 | 22.5 |  |
| ≥80 | 12 | 16.5 | 23 | 17.0 |  |
| Ancestry/ethnicity |  |  |  |  |  |
| European, non-Hispanic | 25 | 55.9 | 56 | 74.0 | 0.07 |
| African, non-Hispanic | 28 | 29.8 | 40 | 19.1 |  |
| Hispanic | 5 | 10.6 | 9 | 6.2 |  |
| Other | 5 | 3.7 | 2 | 0.7 |  |
| Body mass index (kg/m^2^) |  |  |  |  |  |
| <25 | 12 | 22.7 | 16 | 13.3 | 0.27 |
| 25-30 | 15 | 35.8 | 20 | 19.2 |  |
| ≥30 | 25 | 29.1 | 41 | 37.6 |  |
| Unknown | 11 | 12.4 | 30 | 29.9 |  |
| Piccirillo Comorbidity Score |  |  |  |  |  |
| None | 19 | 28.3 | 36 | 35.1 | 0.14 |
| Mild | 25 | 44.5 | 43 | 38.9 |  |
| Moderate | 9 | 12.1 | 20 | 19.2 |  |
| Severe | 10 | 15.2 | 6 | 4.5 |  |
| Unknown | 0 | 0.0 | 2 | 2.3 |  |
| Health insurance |  |  |  |  |  |
| Private | 26 | 40.2 | 51 | 52.1 | 0.03 |
| Medicare only/public | 11 | 14.3 | 27 | 24.5 |  |
| Medicaid | 17 | 33.9 | 22 | 14.4 |  |
| No insurance or unknown | 9 | 11.6 | 7 | 9.1 |  |
| Poverty level (census tract) |  |  |  |  |  |
| <20% below poverty | 34 | 59.8 | 74 | 80.2 | 0.01 |
| ≥20% below poverty | 28 | 39.4 | 32 | 19.4 |  |
| Education level (census tract) |  |  |  |  |  |
| <25% no high school degree | 28 | 52.5 | 55 | 63.8 | 0.27 |
| ≥25% no high school degree | 34 | 46.7 | 51 | 35.8 |  |
| Urbanicity of residential location |  |  |  |  |  |
| Urban | 41 | 72.4 | 53 | 45.3 | 0.001 |
| Rural | 7 | 8.9 | 16 | 9.4 |  |
| Urban/rural mix | 14 | 17.9 | 37 | 44.8 |  |
| Unknown | 1 | 0.8 | 1 | 0.4 |  |

*Percentages weighted based on sampling design.

**P-values from chi-squared tests.

**Supplemental Figure 1**. Survival analysis of IBC patients compared to LABC and all other breast cancer patients based on all-cause (A) and breast cancer-related (B) mortality.

**A**


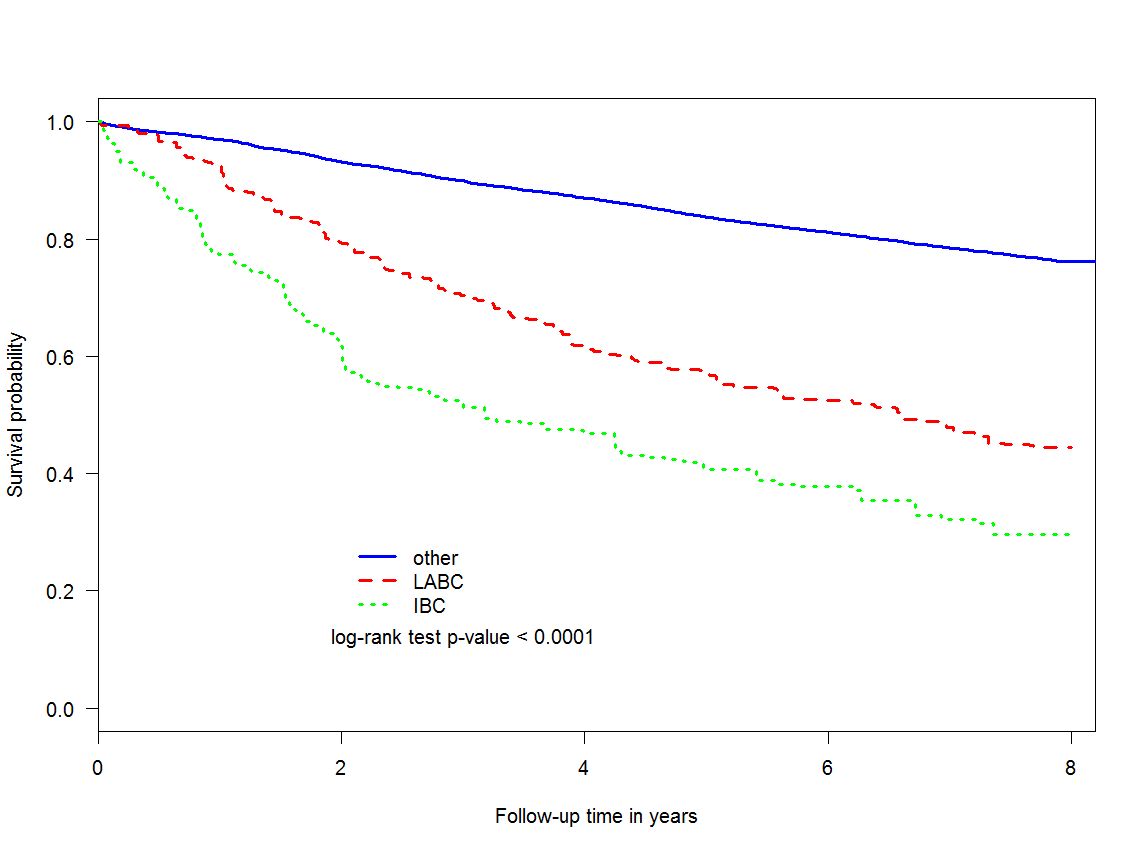


**B**


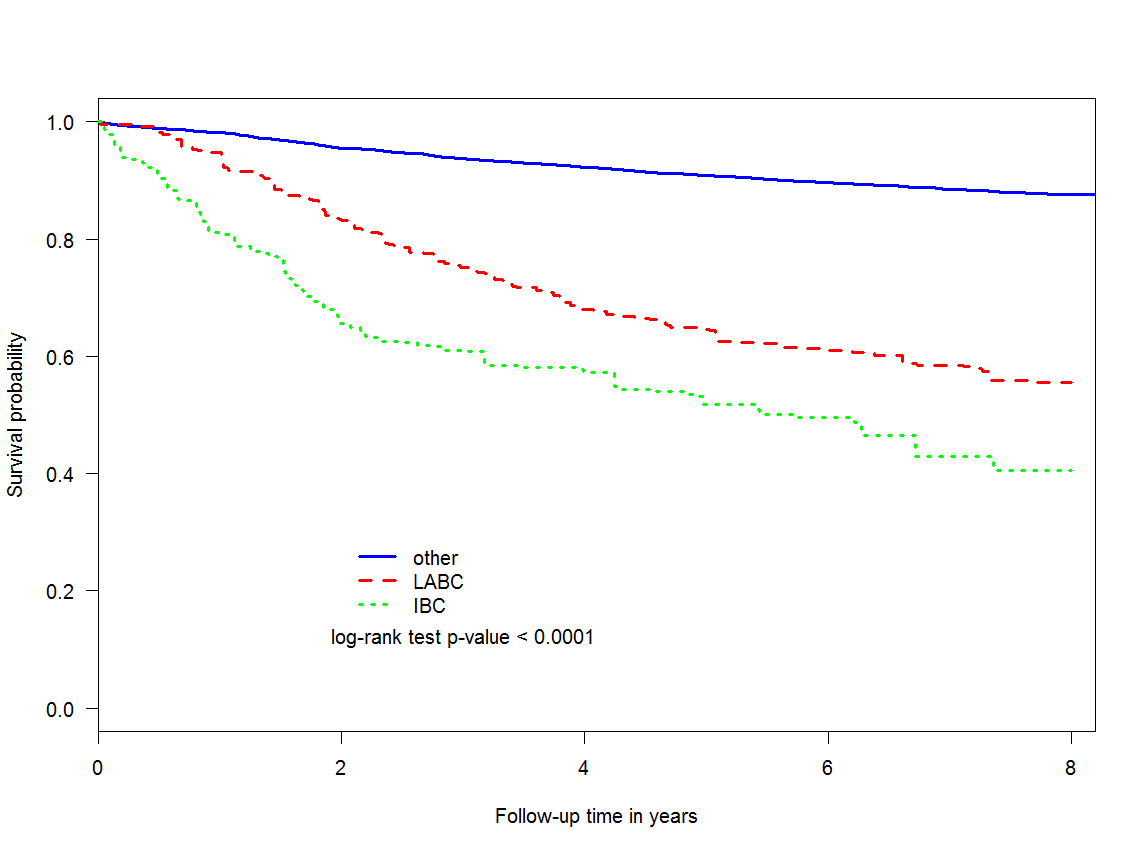
**Supplemental Figure 2**. Comparison of survival among IBC patients based on ER/PR status. Analysis was done based on all cause (A) and breast cancer-related (B) overall survival.

**A**


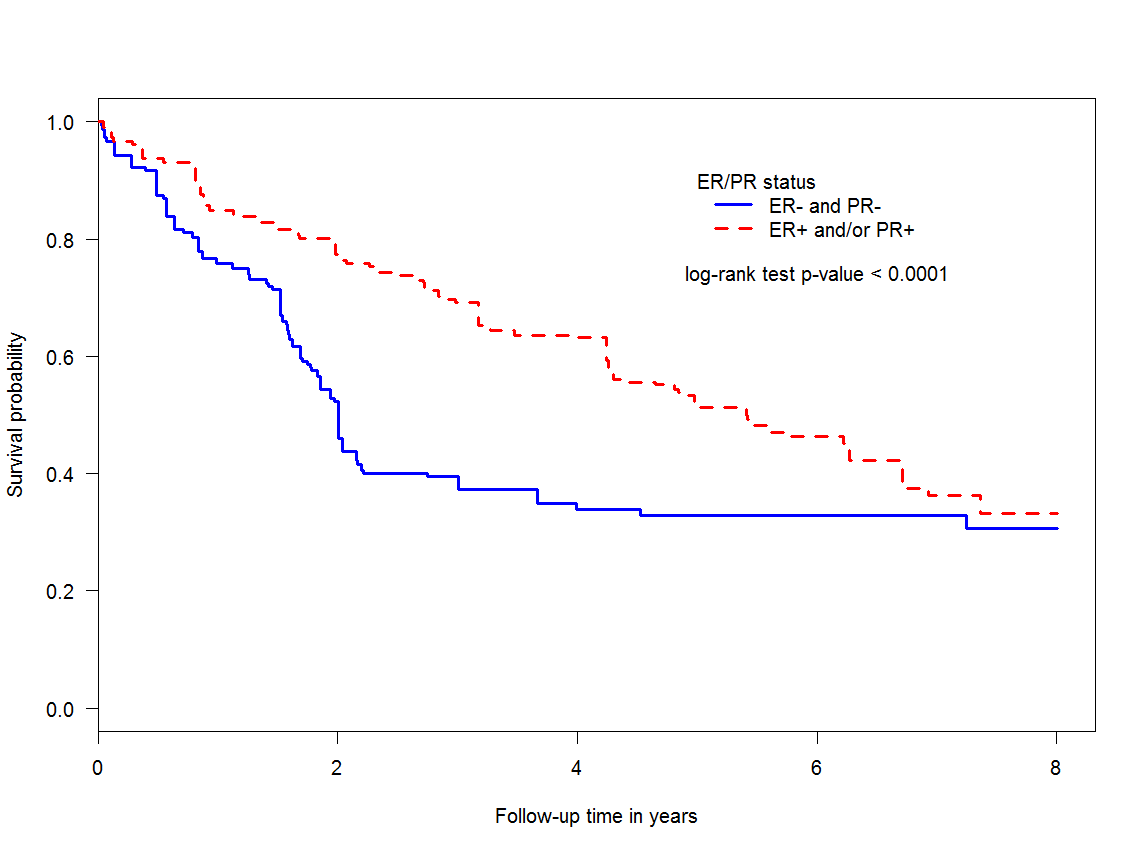


**B**


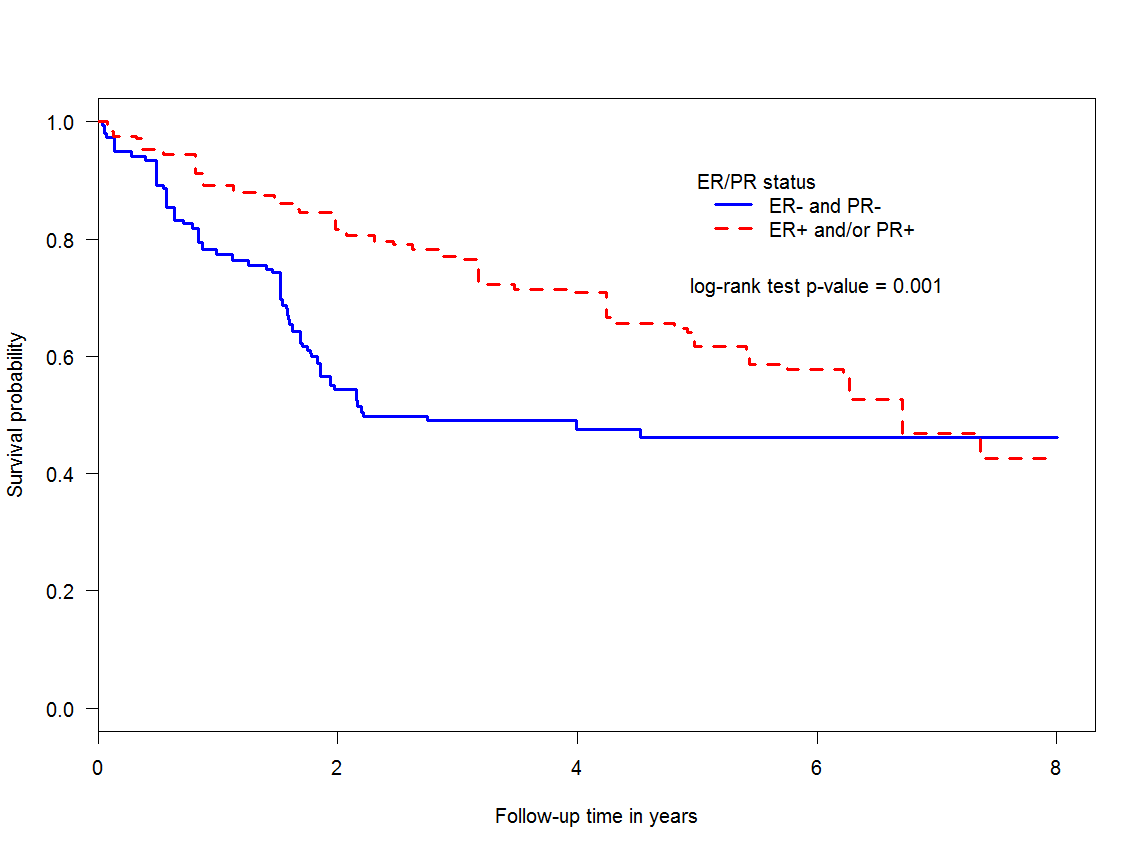


**Supplemental Figure 3**. Survival among IBC patients based on presentation with metastatic versus non-metastatic disease. Analysis was done based on all cause (A) and breast cancer-related (B) overall survival.

**A**


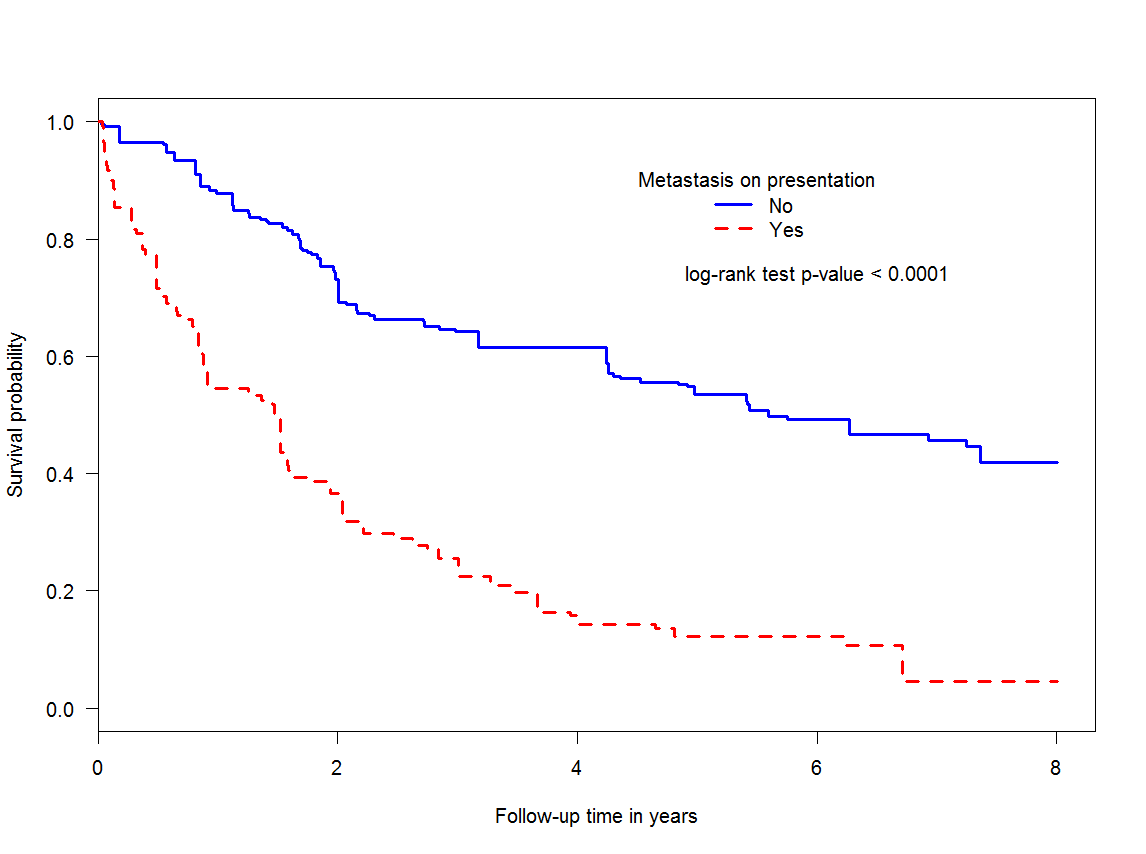


**B**


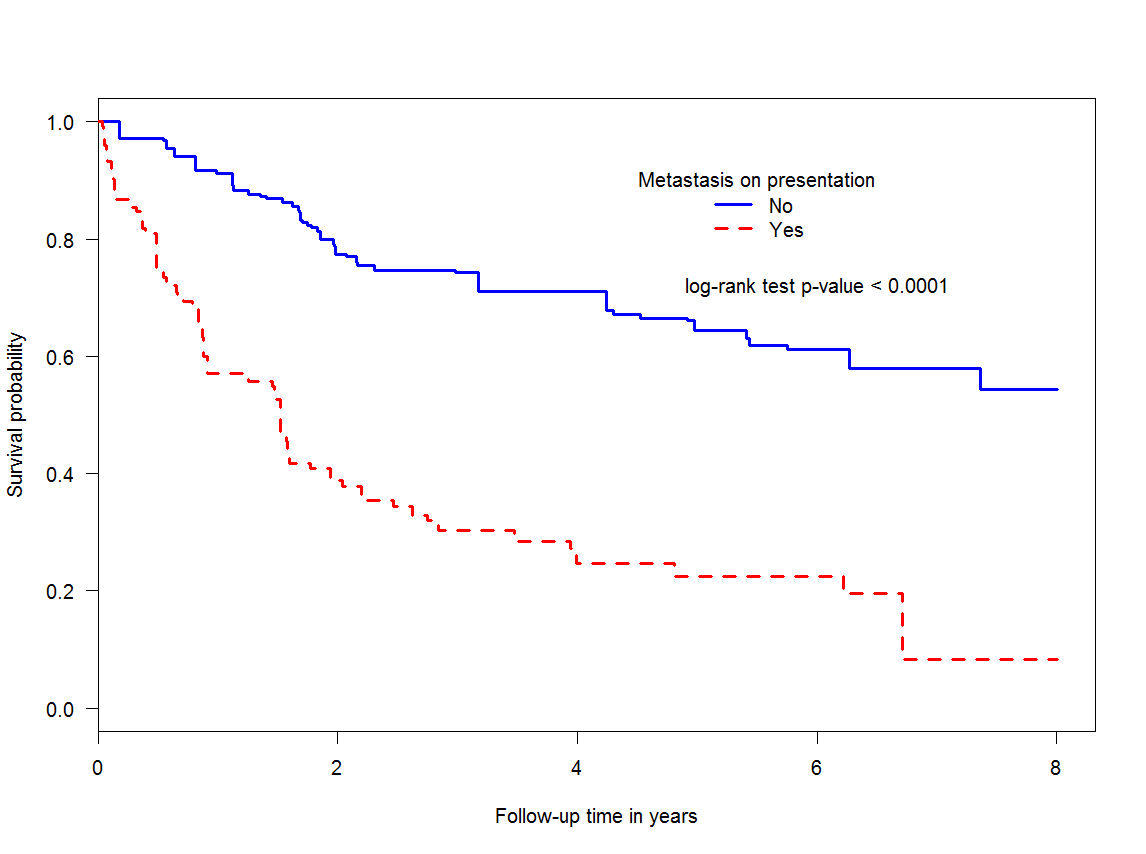

Supplement: Supplementary file 1 — Supplemental Table 1: Characteristics of patients with inflammatory breast cancer according to presentation with metastatic versus non-metastatic disease, Patterns of Care Study, 2004-2012. Supplemental Figure 1: Survival analysis of IBC patients compared to LABC and all other breast cancer patients based on all-cause (A) and breast cancer-related (B) mortality. Supplemental Figure 2: Comparison of survival among IBC patients based on ER/PR status. Analysis was done based on all cause (A) and breast cancer-related (B) overall survival. Supplemental Figure 3: Survival among IBC patients based on presentation with metastatic versus non-metastatic disease. Analysis was done based on all cause (A) and breast cancer-related (B) overall survival. [file 7574946.f1.docx]
